# Investigating the effect of tumor vascularization on magnetic targeting *in vivo* using retrospective design of experiment

**DOI:** 10.1016/j.biomaterials.2016.08.030

**Published:** 2016-11

**Authors:** Kuo-Ching Mei, Jie Bai, Silvia Lorrio, Julie Tzu-Wen Wang, Khuloud T. Al-Jamal

**Affiliations:** a Institute of Pharmaceutical Science, Faculty of Life Sciences & Medicine, King's College London, London, SE1 9NH, United Kingdom; b Division of Imaging Sciences and Biomedical Engineering, King's College London, King's Health Partners, St. Thomas' Hospital, SE1 7EH, United Kingdom

**Keywords:** Enhanced permeability and retention, Immunohistochemistry, Nanocapsules, Nanomedicine, Superparamagnetic iron oxide nanoparticles

## Abstract

Nanocarriers take advantages of the enhanced permeability and retention (EPR) to accumulate passively in solid tumors. Magnetic targeting has shown to further enhance tumor accumulation in response to a magnetic field gradient. It is widely known that passive accumulation of nanocarriers varies hugely in tumor tissues of different tumor vascularization. It is hypothesized that magnetic targeting is likely to be influenced by such factors. In this work, magnetic targeting is assessed in a range of subcutaneously implanted murine tumors, namely, colon (CT26), breast (4T1), lung (Lewis lung carcinoma) cancer and melanoma (B16F10). Passively- and magnetically-driven tumor accumulation of the radiolabeled polymeric magnetic nanocapsules are assessed with gamma counting. The influence of tumor vasculature, namely, the tumor microvessel density, permeability and diameter on passive and magnetic tumor targeting is assessed with the aid of the retrospective design of experiment (DoE) approach. It is clear that the three tumor vascular parameters contribute greatly to both passive and magnetically targeted tumor accumulation but play different roles when nanocarriers are targeted to the tumor with different strategies. It is concluded that tumor permeability is a rate-limiting factor in both targeting modes. Diameter and microvessel density influence passive and magnetic tumor targeting, respectively.

## 1 Introduction

Conventional anti-cancer agents used in chemotherapies do not demonstrate predominant tumor specificity compared to normal cells. Therapeutic effects largely rely on the general systemic biodistribution. Cancer therapy, therefore, is often terminated as a result of the severe side effects, caused by the therapeutic agents, before the expected therapeutic outcome is reached [1]. Nanotechnology *e.g.*, nanoparticle-based drug delivery (nanomedicine), has emerged providing a more efficient targeting to tumors by passive accumulation which is known as the enhanced permeability and retention (EPR) effect [2].

A recent clinical study showed positive tumor accumulation of indium-111 radiolabeled PEGylated liposomes in 15 out of 17 patients employed [3]. The percentage of the injected dose per kg of tumor (% ID/kg) being taken up by the tumors, varied hugely from 2.7% to 53.0% ID/kg at 72 h, among patients with different or even the same type of tumor. The study hypothesized that the considerable heterogeneity in the EPR effect was likely due to the heterogeneous structural and functional integrity of the tumor neovasculature [3].

Magnetic targeting has been proven to enhance tumor accumulation to a higher extent than passive targeting. Increased tumor accumulation of the superparamagnetic iron oxide nanoparticles (SPIONs) can be achieved when a magnetic field is applied to the targeted site [4]. Concentrating the therapeutic agents at the tumor site allows higher doses to be administered at reduced systemic side effects [5]. However, it is expected that not all experimental subjects will benefit from magnetic targeting therapy to the same extent due to such differences in EPR [6]. As a result, in addition to nanoparticle engineering and their physicochemical properties, there are biological factors to consider in the field of passive and magnetic targeting. It is important to identify the potentially important parameters that influence passive and magnetic targeting in different types of tumor.

As suggested in some previous studies, the heterogeneity in tumor vascularization tends to influence passive accumulation of nanocarriers in solid tumors [3], [6]. No study has been performed so far to understand how the vascular factors influence magnetic targeting. In this work, we aimed to understand if tumor vasculature has any influence on magnetic targeting, in comparison to passive targeting, and if so, to identify the key parameters. Four tumor models, of contrasting enhanced permeability and retention (EPR) effect and vascularization characteristics, were used: colon (CT26), breast (4T1), lung (Lewis lung carcinoma) cancer and melanoma (B16F10). Three vascular parameters were chosen, namely, microvessel density, vessel pore size cutoff and vessel diameter. A retrospective design of experiment (DoE) study was conducted using the biodistribution data obtained with gamma counting. The nanocarrier used is a previously developed magnetic nanocapsule (*m*-NCs) combined with passive or magnetic targeting approach. The two main responses studied are %ID/g tumor following passive (TU-) or magnetic (TU+) targeting. The results concluded that these vascular parameters could significantly influence passive and magnetic targeting to various degrees. Furthermore, taking advantage of the DoE approach, it was possible to extract mathematic relationship, *i.e.*, equations, to quantify the impact that each single vascular factor had on passive/magnetic targeting.

## 2 Materials and methods

### 2.1 Materials and reagents

‘Ferrofluid’ magnetic oil (oleic acid coated SPIONs with a diameter of 10 nm, suspended in kerosene at 10^17^ particles per ml) was purchased from Magnacol Ltd (UK). The constituent oleic acid coated SPIONs were separated from the kerosene solvent by precipitation in isopropanol and dried to provide the SPION powder that was subsequently used in the *m*-NC formulations. Soybean lecithin (Epikuron 140 V) was a kind gift from Cargill Pharmaceuticals (USA). Polyoxyethylene-bis-amine (NH_2_-PEG_3.5KDa_-NH_2_) was purchased from JENKEM (USA). D/l-lactide/glycolide copolymer 75/25 (PLGA_18KDa_-COOH) was purchased from Purac Biomaterials (Netherlands). Tween^®^ 80, nitric acid, methanol, dimethylsulphoxide (DMSO) and dichloromethane were obtained from Fisher Scientific Ltd (UK). Sodium chloride, phosphate buffered saline tablets and diethylene triamine pentaacetic acid (DTPA), castor oil and ethylenediaminetetraacetic acid disodium salt dihydrate (EDTA) were purchased from Sigma-Aldrich (UK). Advanced Roswell Park Memorial Institute (RPMI) medium, penicillin-streptomycin 100X, Trypsin-EDTA (1X) with Phenol red, Glutamax™ supplement, phosphate buffered saline PBS (10X, pH 7.4) and phosphate buffered saline PBS (1X, pH 7.4) were obtained from Gibco, Invitrogen (UK). Roswell Park Memorial Institute (RPMI)-1640 and Dulbecco's modiﬁed Eagle's (DMEM) medium were obtained from Sigma Aldrich (UK). Fetal Bovine serum (FBS) was obtained from First-Link Ltd (UK). Disposable square polystyrene cuvettes and disposable capillary cells were purchased from Malvern Instrument (UK). PD-10 desalting column was obtained from GE Healthcare Life Sciences (UK).

### 2.2 Preparation of the *m*-NCs

The magnetic polymeric nanocapsules (*m-*NCs) were prepared by the single emulsification/solvent evaporation method [7]. PEGylated PLGA polymer was synthesized as reported previously [7], [8]. Briefly, PLGA_18KDa_-PEG_3.5KDa_-NH_2_ (12.5 mg), castor oil (75 mg), soybean lecithin (25 mg) and SPIONs (2.5 mg) were dissolved in 2.5 ml dichloromethane. The organic phase was poured into an aqueous phase (5 ml) containing Tween^®^ 80 (20 mg) as a hydrophilic surfactant. The resultant dispersion was emulsified by ultra-sonication using a probe sonicator (Soniprep 150, UK) at 15 micro amplitude for 180 s, in an ice bath. Organic solvents were then evaporated in a chemical fume hood for 20 min. The final volume of the *m-*NC suspension was adjusted to 5 ml. The obtained *m-*NC suspension was further condensed by 20 times, yielding 50 mg/ml of the polymer.

### 2.3 Size and zeta potential measurements

The hydrodynamic size (Z-Average), polydispersity index (PDI) and zeta potential of the products were determined by NanoZS (Malvern Instrument, UK) at 25 °C, using disposable square polystyrene cuvettes (for size and PDI) or disposable capillary cells (for zeta potential). The Z-Average diameter and polydispersity index were measured in water and presented as the average value of three measurements, with 15 runs within each measurement. The zeta potential was also measured in water and presented as the average value of three measurements, with 20–25 runs within each measurement. The mean and standard deviation of size and zeta potential were calculated for each sample.

### 2.4 Determination of SPION encapsulation efficiency in *m*-NCs

*m-*NCs were purified by size exclusion chromatography (G75 column) to remove any non-encapsulated SPIONs. The Fe content was determined by inductively coupled plasma mass spectrometry (Perkin Elmer SCIEX ICP mass spectrometer, ELAN DRC 6100, USA). For ICP-MS measurements, Fe standards (Leeman Labs Inc., MA) were prepared in 20% nitric acid to obtain a standard curve in the range of 10–10000 parts per billion with respect to Fe. *m-*NCs were digested in 2 ml of nitric acid and incubated overnight at 50 °C. The resulting solution was diluted by 10 times in water before measurement.

### 2.5 Radiolabeling of *m*-NCs and serum stability studies

To radio-label the *m-*NCs with indium-111, *m-*NCs were prepared as described above except that PLGA_18KDa_-PEG_3.5KDa_-DTPA was included at 10% (w/w) of the total polymer content. The *m*-NC suspension (50 mg/mL of polymer) was incubated with 2 M ammonium acetate (one-ninth of the reaction volume, pH 5.5), to which 1 MBq of ^111^InCl_3_ (Mallinckrodt, UK) per injection dose was added. The reaction was kept at room temperature for 30 min with intermittent vortexing every 10 min. Upon completion, the radio-labeling reaction was quenched by the addition of 0.1 M EDTA chelating solution (one twentieth of the reaction volume). ^111^InCl_3_ alone was subjected to the same labeling reaction conditions and used as a control.

The *m-*NCs-^111^In were passed through PD-10 columns (size exclusion chromatography) before injecting into animals to exchange the ammonium acetate buffer (pH 5.5) with PBS (pH 7.4) and to remove free ^111^In-EDTA. The *m-*NCs-^111^In (∼150 μL per injection dose, 25 mg/mL of polymer) were collected from the column and spotted on instant thin layer chromatography (iTLC) strips which were then developed in 0.1 M ammonium acetate containing 50 mM EDTA as a mobile phase. Strips were allowed to dry before being developed and counted quantitatively using a cyclone phosphor detector (Packard Biosciences, UK) to ensure that no free ^111^In-EDTA was present in the injected solution.

### 2.6 CD31 immunohistochemistry staining

Murine vascular blood vessels were detected using species-specific CD31 immunostaining. Unstained tumor sections were processed after conventional deparaffinization and endogenous peroxidase quenching using 3% hydrogen peroxide. Tumor tissue sections were incubated with 10% normal donkey serum in PBS for 3 h at room temperature to block non-specific binding sites. Rabbit anti-mouse CD31 primary antibody (ab28364, Abcam, UK) was diluted 1: 50 in 2% donkey serum in PBS and incubated for 3 h at room temperature, followed by incubation with the secondary anti-rabbit HRP using an X-Cell-Plus HRP Detection Kit (Menarini Diagnostics, UK) for 45 min at room temperature. The reaction was revealed using a VECTOR SG Peroxidase (HRP) Substrate Kit (Vector Laboratories, US) for 10 min at room temperature. All sections were counterstained with Neutral Fast Red (Sigma Aldrich, UK).

### 2.7 Microvessel density (MVD) assessment

The microvessel density (MVD) in the tumor was estimated as previously described in the literature [9]. The assessment was performed using Leica DM2000 light microscope (UK) and images were required using a Q-capture pro 7 software. Briefly, immunostained tumor sections were initially examined at low magnification (10X) to locate the highly vascularized areas. Three areas with the highest number of discrete microvessel profiles were selected subjectively as the hot spot areas. One microscopic field was identified within each hot spot area and examined at higher magnification (20X). All individual microvessel profiles within the applied microscopic field were counted according to the generally accepted criteria reported in the literature [9], [10]. These included any stained individual endothelial cell and clusters separated from adjacent microvessels or microvessels with or without vascular lumens. Microvessels in necrotic or sclerotic areas within a tumor and areas adjacent to the invasive carcinoma were not considered in the vessel counts. The MVD was defined as the average number of manually counted vessel of three hot spots per mm^2^ of tumor tissue.

### 2.8 Vessel pore size cutoff (CO) and diameter (DM) assessment

The assessment of CO and DM has been reported in the literature. In the reported studies, CO in the four tumor types was determined by assessing the extravasation and tumor accumulation of the radiolabeled liposomes and lipid micelles with different hydrodynamic sizes. The assessment of CO in CT26/B16F10, 4T1 and LLC tumors was reported by Ishida et al. [11], Charrois et al. [12] and Kashiwagi et al. [13], respectively. The liposomes and lipid micelles were intravenously injected into the tumor-bearing mice and %ID/g of tumor was quantified by measuring the radioactivity. The lower and upper end of the CO range in each type of tumor were determined when a significant increase or reduction in %ID/g of tumor were observed, respectively, upon increasing the size of the nanocarrier.

The mean vessel DM values were previously determined by other groups using CD31 immunofluorescence staining and confocal microscopy. The assessment of DM in CT26, 4T1, B16F10 and LLC tumors was reported by Fisher et al., Goel et al., Bouvard et al. and Braun et al. [14], [15], [16], [17], respectively. The vessels diameters were analyzed with either ImageJ (CT26), an in-house MATLAB algorithm (4T1), Histolab™ software (B16F10) or Fiji software (LLC).

### 2.9 Radiolabeling of *m*-NCs and serum stability studies

To radio-label the *m-*NCs with indium-111, *m-*NCs were prepared as described above except that PLGA_18KDa_-PEG_3.5KDa_-DTPA was included at 10% (w/w) of the total polymer content. The *m*-NC suspension (50 mg/mL of polymer) was incubated with 2 M ammonium acetate (one-ninth of the reaction volume, pH 5.5), to which 1 MBq of ^111^InCl_3_ (Mallinckrodt, UK) per injection dose was added. The reaction was kept at room temperature for 30 min with intermittent vortexing every 10 min. Upon completion, the radio-labeling reaction was quenched by the addition of 0.1 M EDTA chelating solution (one twentieth of the reaction volume). ^111^InCl_3_ alone was subjected to the same labeling reaction conditions and used as a control.

The *m-*NCs-^111^In were passed through PD-10 columns (size exclusion chromatography) before injecting into animals to exchange the ammonium acetate buffer (pH 5.5) with PBS (pH 7.4) and to remove free ^111^In-EDTA. The *m-*NCs-^111^In (∼150 μL per injection dose, 25 mg/mL of polymer) were collected from the column and spotted on instant thin layer chromatography (iTLC) strips which were then developed in 0.1 M ammonium acetate containing 50 mM EDTA as a mobile phase. Strips were allowed to dry before being developed and counted quantitatively using a cyclone phosphor detector (Packard Biosciences, UK) to ensure that no free ^111^In-EDTA was present in the injected solution.

### 2.10 CT26, 4T1, LLC and B16F10 tumor inoculation

All animal experiments were performed in compliance with the UK Home Office (1989) Code of Practice for the Housing and Care of Animals used in Scientific Procedures.

CT26 murine colon adenocarcinoma cells (CT26, ATCC^®^, CRL-2638™) and B16F10 murine melanoma cells (B16-F10, ATCC^®^ CRL-6475™) were cultured in Advanced Roswell Park Memorial Institute (RPMI) 1640 medium. 4T1 murine mammary carcinoma cells (4T1, ATCC^®^, CRL-2539™) were cultured in RPMI-1640 medium. Lewis lung carcinoma cells (LL/2, LLC1, ATCC^®^ CRL-1642™) were cultured in Dulbecco's modiﬁed Eagle's (DMEM) medium. All media were supplemented with 1% Glutamax™, 1% penicillin-streptomycin and 10% fetal bovine serum (FBS). DMEM medium was further supplemented with 1% sodium pyruvate. All cells were cultured in 5% CO_2_ and 95% air at 37 °C.

CT26 and 4T1 were harvested and re-suspended in PBS solution (pH 7.4). 1 × 10^6^ cells in 20 μL were injected subcutaneously and bifocally at the hind foot of syngeneic female BALB/c mice aged 4–6 weeks (Harlan, UK). The same procedures were performed for LLC and B16F10 tumor model in syngeneic female C57Bl6 mice aged 4–6 weeks (Harlan, UK). After inoculation, the tumor volume was measured every other day using a digital caliper and calculated using Equation (1) [18].

(1) $$\text{Tumor}\;\text{volume}\left({\text{mm}}^{3}\right)=\;\frac{4}{3}\text{π}{\left(\frac{A}{2}\right)}^{2}\left(\frac{B}{2}\right)=0.52A^{2}B$$

where *A* and *B* represent the width and the length of the tumors, respectively. All experiment was carried out (*m*-NCs administration) when the tumor volume reached approximately 400 mm^3^–600 mm^3^.

### 2.11 Magnetic targeting setup *in vivo*

Disk-shaped nickel-coated neodymium iron boron (Nd_2_Fe_14_B) magnets (Magnet Expert Ltd, Tuxford, UK) were used for the *in vivo* magnetic drug targeting studies. The magnet was 8 mm diameter, 5 mm thick, N42 grade magnet (product code F324), which had a reported field strength of 0.43 T (T) and a reported ‘vertical pull’ parameter (a measure of the mass of material that the magnet could lift) of 1.9 kg. A single magnet was placed non-invasively over the surface of one of the bifocal tumors and retained using surgical tapes. The contralateral tumor was used as an internal negative control where no magnet was applied. The magnet was then removed at 1 h post-injection of *m*-NCs.

### 2.12 Quantitative organ biodistribution studies by gamma counting

Organ biodistribution, blood circulation and excretion profiles of *m-*NCs-^111^In were assessed quantitatively in tumor-bearing mice by gamma counting. Mice were randomly divided into three groups (*n* = 3) and assigned as 1 h, 4 h, 24 h group. Mice were injected intravenously *via* a lateral tail vein with ∼0.7 MBq *m-*NCs-^111^In (150 μL in PBS). Magnetic targeting was applied as described above. Blood samples (5 μL) were collected from the tail vein at 2 min, 5 min, 10 min, 30 min, 1 h, 4 h and 24 h post-injection. Tumors and other tissues, *i.e.*, skin, liver, spleen, heart, lung, muscle, bone, brain, stomach, intestine, tail and carcass were removed, weighed, and the radioactivity was measured in a gamma counter (1280 CompuGamma Universal Gamma Counter, LKB Wallac, Finland), using the appropriate energy windows for ^111^In. Results were expressed as the percentage of injected dose per gram organ/tumor (% ID/g) as means ± SEM (*n* = 3).

### 2.13 Hematoxylin and eosin (H&E) staining of tissue sections

Portions of major organs *i.e.*, heart, lung, kidney, liver, spleen and tumor tissues were sampled from treated animals, fixed in 10% neutral buffered formalin. Samples were then wax-embedded and sectioned for hematoxylin and eosin (H&E) staining according to standard histological protocols at the Royal Veterinary College, UK. Unstained sections were processed for immunohistochemistry staining as will be described.

### 2.14 Retrospective design of experiment (DoE)

To better understand and visualize which vascular parameters (Factor 1: MVD; Factor 2: CO; Factor 3: DM) may have had an impact on magnetic tumor targeting, a retrospective analysis, using historical biodistribution data in the four murine solid tumor models, was performed. Response surfaces and the predictive equations were then established. Raw data used to create the Predictive Response Surface for Responses 1 (%ID/g TU+) and 2 (%ID/g TU-) are summarized in Table S1. Data were analyzed using Design-Expert 9, v9.0.6.2 (Stat-ease, Inc., USA). Suitable predictive models for Responses 1 and 2 were achieved using Sequential Model Sum of Squares (SMSS). A detailed description of the retrospective DoE analysis is provided in the Supplementary Materials.

### 2.15 Statistical analysis

Quantitative data are presented as mean ± standard deviation (SD) unless specified as mean ± standard error of the mean (SEM). One-way ANOVA and Tukey's multiple comparison test were performed using IBM SPSS version 20. Significance was taken as p < 0.05 unless otherwise stated.

## 3 Results

### 3.1 Preparation and characterization of magnetic nanocapsules *(m*-NCs) for magnetic targeting

The PEGylated oil-cored polymeric magnetic nanocapsules (*m*-NCs) capable of encapsulating high amount of SPIONs were prepared using a single emulsification/solvent evaporation method, as we reported previously [7]. The hydrodynamic diameter obtained for the *m*-NCs is in the range of 200–210 nm with Zeta potential of ∼ −20 ± 1 mV (Table 1). Cryo-TEM images have previously confirmed the core—shell structure of the reported *m*-NCs and showed that the SPIONs are encapsulated within their core [7]. Extensive characterization of *m*-NC has been published by the same authors, and the results demonstrated that this material was prepared successfully. A summary of *m*-NC's characterization is shown in Fig. S1.

### 3.2 Assessment of tumor vasculature characteristics

**Fig. 1:**
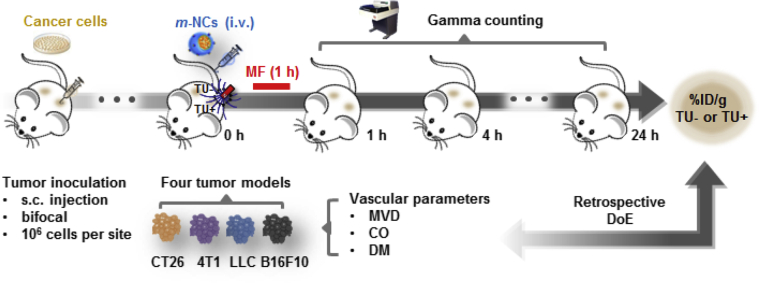
**Schematic description of the experimental design**. Biodistribution assessment of *m*-NCs and vascularization characterization in four murine solid tumor models. CT26 (colon), 4T1 (breast) or LLC (lung), B16F10 (melanoma) cells (1 × 10^6^) were injected subcutaneously (s.c.) and bifocally at the hind foot of female BALB/c (CT26, 4T1) or C57Bl6 mice (LLC, B16F10). Mice were then intravenously (*i.v.*) injected with radiolabeled magnetic nanocapsule, *m*-NC- ^111^In, when tumors reached volumes ∼400–600 mm^3^. A permanent magnet was applied to one tumor for 1 h while the other remained unexposed. The percentage injection dose per gram tumors (%ID/g), with (TU+) or without (TU-) exposure of a magnetic field was assessed with gamma counting. Variables tested are: microvessel density (MVD), vessel pore size cutoff (CO) and vessel diameter (DM).

**Fig. 2:**
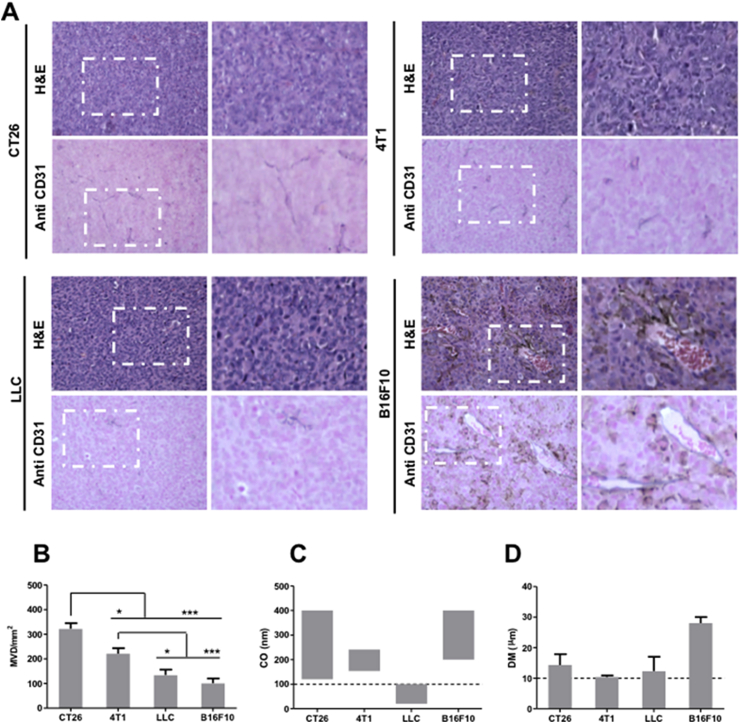
**Tumor vasculature characteristics. (A)** High and low magnification microscopic images of H&E stained and anti-CD31 stained tumor sections (400–600 mm^3^). **(B)** Microvessel density (MVD). **(C)** Average blood vessel's pore size cutoff (CO) and **(D)** diameter (DM), of all tested tumor models. The MVD in tumors was assessed by immunohistochemistry as described in the text. CO and DM values were obtained from Refs. [11], [12], [20] and [14], [15], [16], [17], respectively. For MVD analysis, one-way ANOVA was performed using IBM SPSS version 20 followed by Tukey's multiple comparison test (*p < 0.05 and ***p < 0.005).

Four *in vivo* murine solid tumor models were employed in this study: (i) CT26 colon cancer model (colon carcinoma, fibroblast cells), (ii) 4T1 breast cancer model (mammary gland carcinoma, epithelial cells), (iii) Lewis Lung Carcinoma (LLC) (lung carcinoma) and (iv) B16F10 melanoma (spindle-shaped, epithelial-like cells). The experiment design of this study is illustrated in Fig. 1. The following vascular parameters were assessed in these four tumor models to characterize their tumor vascularization:

**Microvessel density (MVD)**. MVD was assessed by histological examination of CD31 immune-stained tumor sections (Fig. 2a and Fig. S2). CD31 is an endothelial cell marker, characteristic for blood vessels [19]. The MVD values were expressed as the number of blood vessels per mm^2^. As shown in the immunohistochemistry staining images, B16F10 tumors, occasionally pigmented with black/brown melanin granules of different sizes, contained a number of erythrocyte-filled, wide, lumen-containing vessels with clear endothelial linings. CT26 and 4T1 tumors demonstrated much smaller vessel diameters than those observed in B16F10 tumors. MVD values for the different tumors are shown in Fig. 2b. A considerable degree of MVD heterogeneity was found, in the following order: CT26 (322 ± 56) > 4T1 (221 ± 55) > LLC (134 ± 54) ≥ B16F10 (101 ± 48).

Blood vessel pore size cutoff (CO). This parameter is indicative of vessel permeability. CO values reported in the literature for CT26, 4T1 and B16F10 tumor blood vessel pore sizes are in the range of 120–400 nm [11], 154–241 nm [12] and 200–400 nm [11], respectively. LLC tumors, on the other hand, are known to be less permeable, with CO between 20 and 100 nm [20]. CO values are summarized in Fig. 2c.

**Blood vessel diameter (DM)**. The DM of the four tumor types used in the study are reported in the literature and are summarized in Fig. 2d. Values ranged between 10.4 and 14.3 μm for CT26 [14], 4T1 [15] and LLC tumors [17]. A larger vessel DM, up to 28 ± 2 μm, is reported for B16F10 tumors [16].

### 3.3 Evaluation of blood circulation and organ distribution *in vivo*

Tumor accumulation of *m*-NCs in the four murine solid tumor models was assessed following intravenous (*i.v.*) administration using our previous magnetic targeting protocol [7]. PLGA_18kDa_-PEG_3.5kDa_-DTPA (10% w/w) was incorporated into *m*-NCs, which was then radiolabeled with the gamma emitter, ^111^In, to allow quantitative uptake studies in mice. Blood circulation, excretion, organ biodistribution and tumor accumulation profiles were evaluated and compared by gamma counting. Organ uptake was expressed as the percentage of injected dose per gram of tissue (% ID/g).

**Fig. 3:**
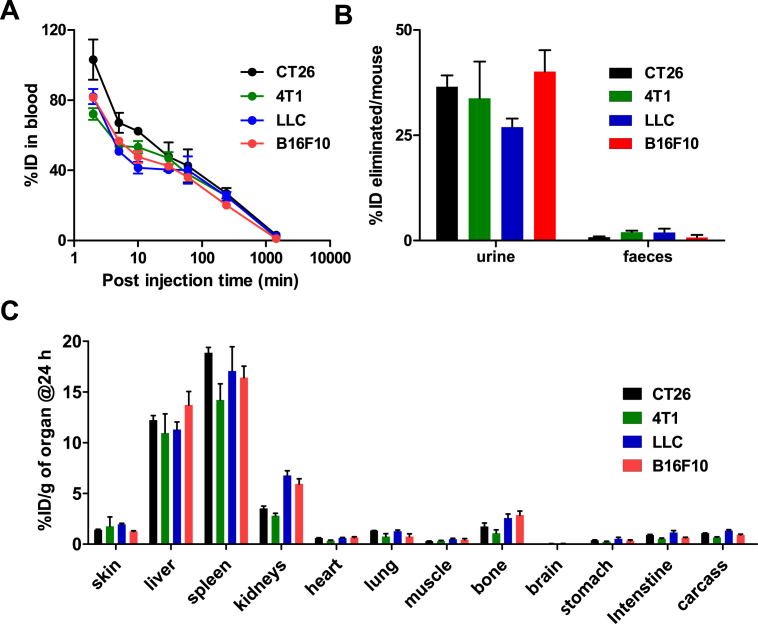
**Organ biodistribution studies of radiolabeled magnetic nanocapsules in major organs following intravenous administration**. Bifocal CT26, 4T1, LLC and B16F10 tumor-bearing mice were intravenously injected with *m*-NC-^111^In, at a dose of 312.5 mg polymer/kg, 125 mg SPION/kg (70 mg Fe/kg). A permanent magnet (0.43 T, 8 mm in diameter) was applied to one tumor for 1 h. Mice were sacrificed at the specified time point, following whole body saline perfusion. **(A)** Blood clearance profiles, **(B)** Excretion profiles and **(C)** Organ biodistribution profiles, at 24 h post-injection. Results are expressed as mean ± SEM (*n* = 3).

Blood circulation profiles are shown in Fig. 3a. Expectedly, *m*-NCs exhibited comparable and prolonged blood circulation in all types of tumor-bearing mice, due to PEGylation of *m*-NCs. Values of ∼36–42%, 20–27% and 1–3% ID in blood were detected at 1, 4 and 24 h post-injection, respectively. About 40% and 0–2% ID/mouse were excreted in urine and feces, respectively (Fig. 3b). Similarly, no differences in organ biodistribution, except tumors, were observed at 24 h post-injection (Fig. 3c). Earlier time point studies (1 and 4 h) are shown in Fig. S3.

Confirming our previous findings in CT26 tumor-bearing mice [7], spleen (14–19% ID/g), liver (11–14% ID/g) followed by the kidney (3–7% ID/g) showed the highest uptake (Fig. 3c). These results suggest that *m*-NCs exhibited matching blood circulation, elimination and major organ biodistribution profiles in all tumor-bearing mice tested, so any differences in tumor uptake profiles are likely to be tumor type-dependent.

### 3.4 Passive and magnetic targeting of solid tumors *in vivo*

**Fig. 4:**
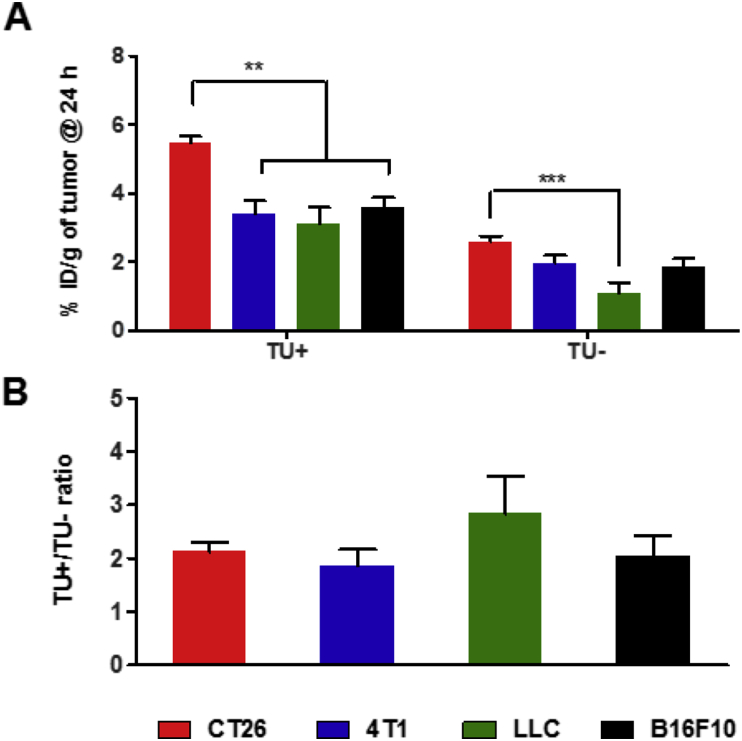
**Tumor uptake of radiolabeled magnetic nanocapsules in solid tumors following intravenous administration**. Bifocal CT26, 4T1, LLC and B16F10 tumor-bearing mice were intravenously injected with *m*-NC-^111^In, at a dose of 312.5 mg polymer/kg, 125 mg SPION/kg (70 mg Fe/kg). A permanent magnet (0.43 T, 8 mm in diameter) was applied to one tumor (TU+) for 1 h. The contralateral tumor remained unexposed (TU-) and was used as a baseline control, for each of the tumor types tested. Mice were sacrificed at the specified time point, following whole body saline perfusion. **(A)** The percentage injection dose per gram tumors (%ID/g), with (TU+) or without (TU-) exposure of a magnetic field, was assessed with gamma counting, at 24 h. **(B)** Magnetic drug targeting, quantified by the fold increase in tumor uptake upon application of magnetic field, expressed as TU+/TU- ratio. Results are expressed as mean ± SEM (*n* = 3). One-way ANOVA was performed using IBM SPSS version 20 followed by Tukey's multiple comparison test (**p < 0.01 and ***p < 0.005).

Magnetic targeting efficacy in the tumors was expressed as fold increase (TU+/TU- ratio) in % ID/g tumor, upon the application of an external magnetic field (magnetic targeting, TU+), compared to non-magnetically targeted tumors (passive targeting, TU-). The absolute tumor uptake values obtained at 24 h varied significantly between the different tumor types, under both passive and magnetic targeting conditions (Fig. 4a). Values at 1 and 4 h are presented in Fig. S4. Higher % ID/g tumor values were found for magnetically targeted (TU+) than passively targeted tumors (TU-) in all tumor types tested. In case of passive targeting, the following order in tumor uptake was obtained: CT26 (2.6 ± 0.2%) ≥ 4T1 (2.0 ± 0.2%) ≥ B16F10 (1.8 ± 0.2%) > LLC (1.1 ± 0.3%). For magnetically targeted tumors (TU+), the following order and values were obtained: CT26 (5.7 ± 0.2%) (*p* < 0.05) > B16F10 (3.6 ± 0.3%) ≥ 4T1 (3.4 ± 0.4%) ≥ LLC (3.1 ± 0.5%). The TU+/TU- ratios ranged between 1.8 ± 0.3 and 2.8 ± 0.7 in all tumor models tested (*p* > 0.05) (Fig. 4b). It is worth noting that results presented here were performed following the whole body saline perfusion so it is unlikely that the tumor uptake data are influenced by blood contamination.

Our results confirmed that magnetic targeting does not only improve uptake in highly vascularized (↑MVD) and permeable (↑CO) tumors, *e.g.*, B16F10 tumors but poorly vascularized and less permeable tumors, *e.g.*, LLC tumors, can also benefit from this targeting approach.

### 3.5 Retrospective design of experiment (DoE) analysis

To identify the key vascular characteristics and visualize the impact of changing vascular multiple parameters on magnetic tumor targeting, we have applied a retrospective design of experiment (DoE) analysis using historical biodistribution data, to establish the response surfaces and the predictive models. Further details on DoE studies are in the Supplementary Materials (Table S1-15 and Fig. S5, Fig. S6). We identified three quantitative factors, MVD in number/mm^2^ (Factor A), mid-point CO values in nm (Factor B) and mean DM in μm (Factor C), as critical tumor vascularization features to evaluate two responses including %ID/g of TU- (response 1) %ID/g of TU+ (response 2).

**Fig. 5:**
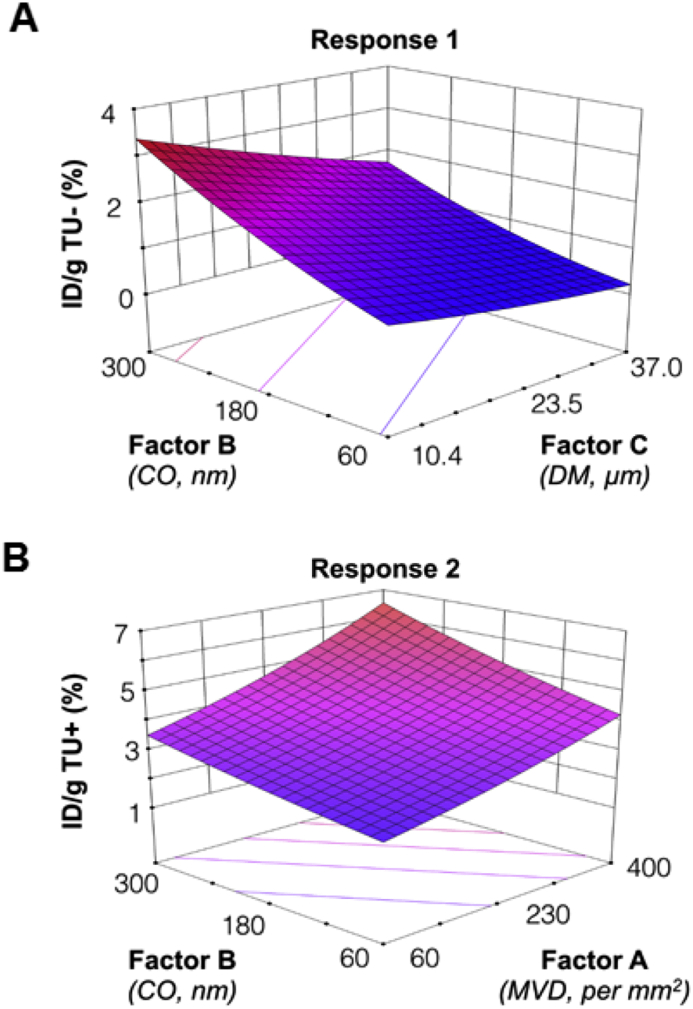
**Predicting the influence of the tumor vascular parameters on magnetic targeting with the retrospective design of experiment (DoE) analysis approach**. Factors tested are factor A: microvessel density (MVD), factor B: vessel pore size cutoff (CO) and factor C: and vessel diameter (DM). Responses are Response 1: %ID/g TU- and Response 2: %ID/g TU+). **(A)** and **(B)** show Predictive Response Surface for Response 1 and Response 2, respectively. The DoE analysis was performed using Design-Expert 9, v9.0.6.2 software.

A linear predictive model of the vascular factors on response 1 was found and their mathematical relationship is presented in Equation (2) and Equation (3), in the coded and actual unit, respectively. The factors were coded by a linear transformation from their original measurement scale to a unified unit to allow direct comparison between factors with different units. Equation (2) demonstrated a positive coefficient of 0.43 and a negative coefficient of −0.26 for CO and DM, receptively, indicating larger CO and smaller DM would favor the passive tumor accumulation. The higher value of the coefficient of CO also suggests that CO is the rate-limiting step for passive tumor uptake, possibly *via* influencing extravasation. The effect of DM appears to be more pronounced in more permeable vessels (CO ∼ 300 nm) than the less leaky ones (CO ∼ 60 nm) in the surface response plot of response 1 (Fig. 5a).

(2) $$\sqrt{ID/g\left(TU-\right)\;}=1.14+0.43B-0.26C$$

(3) $$\sqrt{ID/g\;\left(TU-\right)}=0.95221+\left(0.0361277\times CO\right)-\left(0.019607\times DM\right)$$

In the case of response 2 (magnetic targeting), the significantly related vascular factors were factor A (MVD) and factor B (CO), as shown in Equation (4) and Equation (5) in the coded and actual unit, respectively. Their linear coefficients were both positive, with 0.31 and 0.22 for MVS and CO, respectively. This indicates that higher MVD and CO values encourage the accumulation of *m*-NCs in tumors when a magnetic field is applied (Fig. 5b).

(4) Ln[ID/g(TU+)]=1.33+0.31A+0.22B

(5) Ln[ID/g(TU+)]=0.58464+(0.0181518×MVD)+(0.00182389×CO)

## 4 Discussion

Nanocarriers exploit the EPR effect to passively accumulate in tumors. However, the degree of EPR effect can vary from one tumor to another, or even spatially and temporally within the same tumor [21]. This heterogeneity in the EPR effect results in differences in therapeutic response rates [22]. Tumors that are known to be extensively vascularized and permeable, such as head and neck cancer, show high therapeutic response rate to Caelyx^®^ (ALZA Corporation), a liposomal doxorubicin formulation [3], [23]. In contrast, poor therapeutic efficacy was observed in hepatocellular carcinoma treated with a polymer-doxorubicin conjugate [24]. These studies suggested that the heterogeneous tumor uptake of nanocarriers was likely related to the differences in the structural and functional integrity of the tumor neovasculature [3], [21]. Vascular parameters, such as tumor angiogenesis, vessel permeability and perfusion, therefore, can lead to a distinct loco-regional distribution of nanocarriers in the tumor. Harrington et al. showed that the tumor volume could affect the degree of liposomal passive accumulation in patients with squamous cell carcinoma of the head and neck (SCCNH), lung or breast cancers, which was likely due to the presence of poor vascularized or even necrotic areas in the tumor [3]. Koukourakis et al. further confirmed that there was a linear regression between the passive tumor uptake and microvessel density in six non-small cell lung cancer patients [23]. Hobbs et al., on the other hand, suggested that tumors also have tumor-dependent functional pore size cutoff and macromolecule permeability. The transvascular transport of nanoparticles with sizes larger than pore size cutoff can be significantly restricted [25]. In addition to passive targeting, differences in magnetic targeting response were also observed *in vivo* in different experimental subjects and Schleich et al. attributed this effect to the variabilities of tumor vascularization among individuals [6].

Notably, no report today has studied in details the effect of multiple vascular parameters on tumor accumulation, especially with magnetic targeting. Since the first liposomal anti-cancer formulation became available on the market, many types of nanocarriers have achieved outstanding *in vitro* and *in vivo* preclinical performance, have failed the clinical trial stage. This work aims to understand which tumor vascular parameter(s) can influence the degree of nanocarriers uptake in tumors, under both passive and magnetic targeting conditions. This has been achieved by performing pre-clinical *in vivo* biodistribution studies, of a previously reported magnetic-responsive nanocarrier, in mice bearing different types of tumors. The experimental data was then combined this with the DoE approach, to generate mathematical equations. These mathematical equations can assess, in a parameter by parameter fashion, the direct link between each of the vascular factors and the response, in this case, tumor uptake.

Our study showed comparable organ biodistribution, blood circulation and excretion of *m*-NCs in the four murine solid tumor models. Distinct tumor accumulation profiles, under both passive and magnetic targeting conditions, were however obtained. The differences in vascularization of tumor tissues are likely to have influenced the tumor accumulation of *m*-NCs. Tumor angiogenesis is related to high microvessel density (MVD) and it is one of the major abnormal vascular characteristics of tumor tissues [26]. MVD plays a prognostic role in determining cancer malignancies such as metastatic rate [27], tumor angiogenesis [28] and even clinical therapeutic outcomes [29], [30], [31]. This is due to the fact that differences in tumor MVD, as a result of drastic differences in the number of blood vessels, can lead to differences in nanocarrier distribution and accumulation in tumors. MVD counting has been the standard approach for several decades for vascular analysis. The MVD was assessed by immunohistochemistry staining in our study with variable degrees of MVD observed among the four tumor models tested. CT 26 showed the highest MVD which was 1.5, 2.4 and 3.2-fold higher than the MVD values observed in 4T1, LLC and B16F10 tumors, respectively.

Tumor vessel permeability is another parameter considered in this study since it can remarkably affect the rate of cancer growth, predisposition to metastasis and delivery of macromolecular therapeutics to tumor cells [32]. Tumor vasculature is reported to be leaky and permeable with endothelial defects. There is a minimal vessel pore size cutoff (CO) required for nanocarriers to extravasate. Hobbs et al. demonstrated that the pore sizes of tumor blood vessels were highly dependent on the tumor model type and the anatomical location of tumor inoculation. Most murine tumors inoculated subcutaneously exhibited a pore size cutoff ranging between 380 and 780 nm (as large as 1.2–2 μm in one tumor type) [25]. Tumors with small CO tend to have a very limited permeability to macromolecules and consequently low extravasation of nanocarriers. In the four murine solid tumor models tested here, the blood vessels in LLC tumors were significantly less permeable (3–5-fold smaller CO, mid-point values) than in other types [11], [12]. Weissig et al. observed that micelles with 20 nm achieved higher accumulation in LLC tumor compared to liposomes of 100 nm in size, despite the longer blood circulation time of the latter. This agrees with our findings here that LLC tumors also showed the least uptake in passive and magnetically targeted.

Another important vascular parameter in passive delivery is the vessel diameter (DM). DM affects tumor tissue perfusion and blood flow distribution, thus, can potentially affect drug delivery and the therapeutic outcomes [33]. For example, large DM results in reduced vascular surface to plasma volume ratio which therefore reduces the possibility of nanocarriers to reach and pass through the blood vessel wall [34]. Previous studies suggested that blood vessels of B16F10 tumors are 2–3-fold larger than vessels of the other three models [13], [14], [15], [17]. Our histological observations are in line with the reported results where B16F10 melanoma tumors showed vessels with the large lumen, filled with erythrocytes. One, therefore, may expect that the larger diameter of blood vessels in B16F10 tumors should theoretically result in significantly smaller %ID/g for passively targeted B16F10 than CT26 tumors. The gamma counting showed no significant differences in %ID/g TU- between these two models. This could be due to the highest permeability reported for B16F10 tumors compared to all other tumor types tested (mid-point CO values: B16F10 > CT26 > 4T1 > LLC).

In order to single out the effect that each of the vascular parameters may have had on passive and magnetic targeting, tumor uptake values obtained in our study with gamma counting *i.e.*, responses, were fitted into a retrospective DoE analysis. CO has been shown as the rate limiting step in both targeting modes. This is already known for passive targeting. Magnetic dragging forces can enhance passive targeting further by concentrating the nanocarriers within the tumor and increase their possibility of extravasation. It is logical that they are still unable to extravasate if the blood vessels are not leaky enough [35].

The DoE analysis for magnetic targeting (Response 2) indicated that the accumulation of *m*-NCs in tumor tissues, under the influence of an external magnetic field, was also significantly affected by MVD, in a positive manner. CT26 tumors exhibited highest MVD and highest TU + uptake. The high local MVD provides a higher total surface area available for extravasation of nanoparticles into tumor interstitium [36], [37], [38], [39]. Interestingly, DM has not negatively influenced magnetic targeting (p > 0.05). The effect of large DM could have been overcome by the local increase in the nanocarrier concentration within the tumor vasculature, due to the acting magnetic forces, thus compensating for the loss in the vascular surface area available to extravasation.

As the DoE results imply, the tumor vasculature factors which appeared to be significant, *i.e.*, can influence %ID/g, were different between passive and magnetic tumor targeting; CO and DM were significant factors for passive targeting (Table S2, Equations (2), (3)), while MVD and CO were significant factors for magnetic targeting (Table S9, Equation (4), (5).

Patient selection, based on their tumor vasculature characteristics, can become an important factor to consider when selecting patients for magnetically targeted therapies. In passive tumor targeting, for example, larger CO results in higher %ID/g whereas the DM can inversely, but mildly, affect %ID/g (Equation (2)). LLC tumor has the lowest CO which resulted in the lowest %ID/g TU- compared to other tumor types. CT26 and B16F10 have large CO but CT26 has a smaller DM than B16F10 hence CT26 achieved the higher %ID/g TU- than B16F10 and also other tumors.

When the magnetic field was applied, MVD appears to be the major contributing factor towards %ID/g tumor, with CO comes second (Equation (4)). The true value coefficient for MVD is ∼10 times higher than CO (Equation (5)). LLC tumor, for example, has a moderate MVD value and managed to achieve a 4-fold increase in tumor uptake with magnetic targeting, *i.e.*, TU+/TU- ratio (Fig. 4), despite its lowest CO value. The DoE provides a good rationale why LLC achieved the highest improvement with magnetic targeting despite its lowest %ID/g TU-. Overall, the DoE results provide a mathematical tool to predict optimal therapeutic outcomes, using magnetic targeting, knowing the tumor vasculature characteristics of the solid tumor to be treated. Another positive insight from this data is that even tumors with low CO but of large DM, and moderate-to-high MVD are very likely to benefit from magnetic targeting based therapy.

In addition to biological factors, one may propose that the effect of physical parameters such as nanoparticle size among others is interesting to study using the DoE approach. It is however conceivable that such parameters are likely to alter NP's pharmacokinetic profiles and interactions with the tumor, introducing multi-factorial interactions. This type of experiments involving multi-factorial interactions can be combined with DoE approach but requires a new experimental design altogether and cannot be done retrospectively using this set of data. On this relevant topic and in a very recent work by our group [40], [41], we have performed extensive studies, not involving DoE design, investigating the effect of increasing SPION loading of the nanocapsule, while maintaining the same nanocapsule size, on blood circulation time, organ biodistribution and tumor targeting in CT26 tumor model. A linear increase in magnetic targeting as a function of increased SPION dose was found. No difference in organ biodistribution or blood circulation time was observed among the *m*-NC formulations, containing different amounts of SPION, up to 125 mg/kg dose, a dose above which an increased liver and spleen uptake and reduced blood circulation times started to be observed. Magnetic targeting efficiency plateaued at that SPION dose. The stability of our formulation in presence of serum has also been established [41].

## 5 Conclusions

The novelty of this work relies on investigating the effect of biological parameters of solid tumors, on magnetic targeting, which has only been studied in the context of passive tumor targeting to date. In addition, the DoE approach was used to extract mathematical relationship to quantify the impact that each single vascular factor had on passive/magnetic targeting. These studies are important as they shed light on which tumor types are likely to be suitable for magnetic targeting therapy. We have proven here that the important biological factors can be different in magnetic targeting from those reported to be relevant to passive tumor targeting.

Our study concerned establishing a correlation between tumor vascular parameters and degree of tumor uptake following passive and magnetic targeting. The results suggested that CO was a rate limiting factor in both targeting modes. DM and MVD influenced passive and magnetic targeting, respectively. Our study demonstrated in mathematical terms that the influence of an individual vascular parameter had on the passive and magnetic targeting of magnetic nanocarriers to tumors. In addition, the findings of this study further confirmed the importance of taking the tumor vasculature characteristics into account when designing nanocarriers for both passive and magnetic targeting. In relevance to clinical applications, our results confirmed that the magnetic targeting approach can be applied to both leaky and less leaky tumors.

## Figures and Tables

**Table 1:** Physicochemical characterization of PEGylated NCs prepared by single emulsification/solvent evaporation method.

Sample	Initial SPION loading^a^	Hydrodynamic size ± SD (nm)^b^^,^^e^	PDI ± SD^e^	Zeta potential ± SD (mV)^c^	SPION EE% ± SD^d^^,^^e^
NCs	–	203 ± 4	0.12 ± 0.01	−45 ± 2	–
*m*-NCs	1.84%	205 ± 3	0.16 ± 0.01	−36 ± 1	95 ± 3

a Values were expressed as w/w SPION/NC. Total NCs weight is calculated by the addition of polymer, lecithin, castor oil, SPIONs and Tween 80^®^ weights.

b Size was measured with dynamic light scattering in deionized water (*n* = 3).

c Values were obtained with laser Doppler electrophoresis and measured in deionized water.

d Iron content was determined with ICP-MS (*n* = 3).

e Results are expressed as mean ± SD (*n* = 3).
