# Supplementary material for: Investigating the effect of tumor vascularization on magnetic targeting in vivo using retrospective design of experiment
Source: Biomaterials. 2016 Nov;106:276–85. doi: 10.1016/j.biomaterials.2016.08.030 (PMC5027889; doi:10.1016/j.biomaterials.2016.08.030)
Supplement: Supplementary file 1 [file mmc1.pdf]

Supplementary Data

Investigating the Effect of Tumor Vascularization on Magnetic Targeting *In Vivo* Using Retrospective Design of Experiment

Kuo-Ching Mei[a†], Jie Bai[a†], Silvia Lorrio[b], Julie Tzu-Wen Wang[a], Khuloud T. Al-Jamal[a]*

[a]Institute of Pharmaceutical Science, Faculty of Life Sciences & Medicine

King's College London, SE1 9NH (UK)

[b] Division of Imaging Sciences and Biomedical Engineering, King’s College London,

King’s Health Partners, St. Thomas’ Hospital, SE1 7EH, United Kingdom

[†] Contributed equally to the work

* Corresponding-Author

Dr Khuloud T. Al-Jamal

Institute of Pharmaceutical Science, Faculty of Life Sciences & Medicine

King's College London, SE1 9NH (UK)

E-mail: khuloud.al-jamal@kcl.ac.uk

Supplementary Methods

Developing response surface from historical data analysis

Four different solid tumor models *i.e.*, CT26 (colon), 4T1 (breast), LLC (lung), and B16F10 (melanoma) were used to evaluate the tumor accumulation of the *m*-NCs, with or without the application of a magnetic field. To better understand and visualize which vascular factors (MVD, CO or DM) may have had an impact on magnetic tumor targeting, a retrospective DoE analysis, using historical data, was performed to establish the response surfaces and the predictive model. Raw data that are used to create the Predictive Response Surface for Responses 1 and 2 are summarized in **Table S1.** Data were analyzed using Design-Expert 9, v9.0.6.2 (Stat-ease, Inc., USA). Suitable predictive models for **Responses 1** and **2** were achieved using Sequential Model Sum of Squares (SMSS).

Selecting predictive model using sequential model sum of squares (SMSS)

For Response 1, Box-cox transformation was performed for the raw data to improve model fit (power transformed $\lambda = 0.5$). Step-wise regression (involving forward selection, backwards elimination, and bidirectional elimination) was used to determine the model terms (Alpha in and Alpha out = 0.1). The SMSS was used to select a suitable predictive model for data analysis. The mean square of the model was firstly calculated followed by the addition of a higher level source of term, *i.e.*, a higher degree of the polynomial in the predictive equation. The aim was to include a higher level source of terms only if this could explain a significant amount of variation in the responses when compared with the lower-level model. In other words, when one or more predictor variables (source of term) are included in the model, the error sum of squares (SSE) should be reduced or the regression sum of square (SSR) should be increased. As shown in **Table S2**, the Model F-value of 37.88 implied the model was significant (p-value < 0.0001) and factor B (CO)

and C (D) were significant model terms (p-value < 0.0001). The SMSS table for Response 1 is shown in **Table S3**. Linear predictive model explained a significant amount of variability in the responses when compared to the overall sample mean (p-value <0.001). Adding the two-factor interaction (2FI) into the model did not explain the rest of the variability *i.e.*, no improvement in the model. Linear model was therefore suggested for Response 1. The provisional models were then evaluated with a lack of fit test. The linear model “lack of fit F value” was 7.97, p-value = 0.2727, which implied that there was a 27.27% chance that the lack of fit F value could have occurred due to noise, *i.e.*, the model had a good fit (**Table S4**). Finally, all models were assessed by an overall standard deviation of the model, various R^2 and Predicted Residual Sum of Square (PRESS) statistics (**Table S5**). The "Predicted R^2" of 0.7129 was in reasonable agreement with the "Adjusted R^2" of 0.7623, *i.e.*, the difference was less than 0.2. "Adeq. Precision" of 13.494 indicated an adequate signal, *i.e.*, a good signal-to-noise ratio. The fit test summary for Response 1 is shown in **Table S6**. This model can be used to navigate the design space (**Table S7**). The coefficient of the model terms is shown in **Table S8** with a small Variance Inflation Factor (VIF) of 1.04 for both Factor B and C. The normality of the residual from the predictive model was tested and the results are shown in **Figure S4**, which appeared to be normally distributed.

For Response 2, Box-cox transformation was performed for the raw data to improve model fit (power transformed $\lambda = 0$). Step-wise regression (involving forward selection, backwards elimination, and bidirectional elimination) was used to determine the model terms (Alpha in and Alpha out = 0.1). As shown in **Table S9**, the Model F-value of 21.97 implied that the model was significant (p-value < 0.0001). Factor A (MVD) and Factor B (CO) were significant model terms with a p-value of 0.0003 and 0.0005, respectively. The SMSS table for Response 2 is shown in **Table S10**. Linear predictive model explained a

significant amount of variability in the responses when compared to the overall sample mean (*p*-value <0.001). Adding the two-factor interaction (2FI) into the model did not explain the rest of the variability significantly, *i.e.*, no improvement in the model. Linear model was therefore suggested for Response 2. The linear model lack of fit F value for Response 2 was 15.01, *p*-value = 0.2010, which also indicated a good fit (**Table S11**). The "Predicted R^2" of 0.5951 was in reasonable agreement with the "Adjusted R^2" of 0.6458, *i.e.*, the difference is less than 0.2. "Adeq. Precision" of 13.631 indicates an adequate signal, *i.e.*, a good signal-to-noise ratio (**Table S12**). The fit test summary for Response 2 is shown in **Table S13**. This model could be used to navigate the design space (**Table S14**). The coefficient of the model terms is shown in **Table S15** with a small Variance Inflation Factor (VIF) of 1.04 for both Factor A and B. The normality of the residual from the predicted model was tested and the results are shown in **Figure S5**, which appeared to be normally distributed.

Supplementary Figures

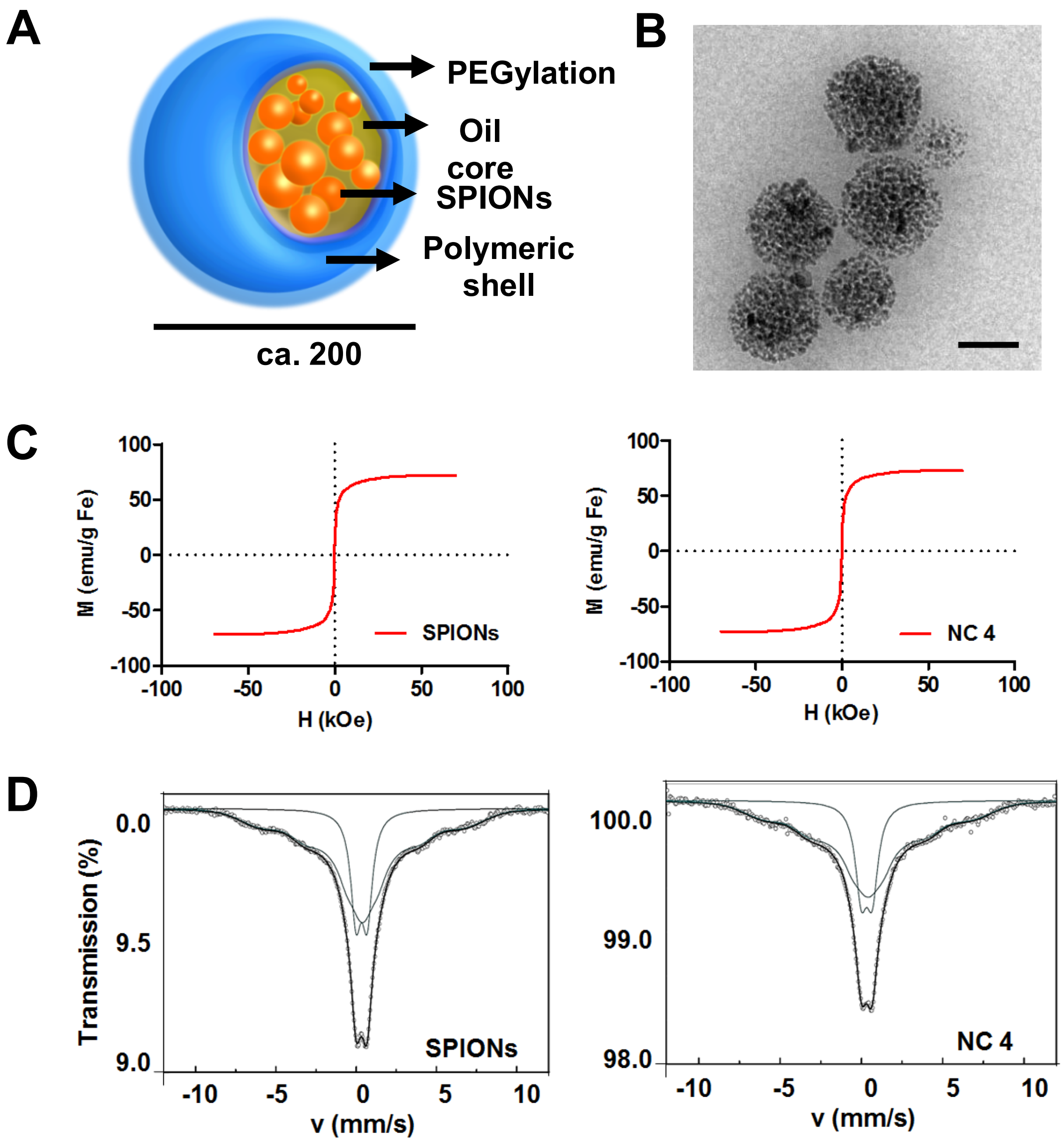

Figure S1. Physiochemical characterization of *m*-NCs. *m*-NC, referred to in this study is the same as NC4 in Al-Jamal *et al*, Nano Letters, 2016). *m*-NCs were prepared by the single emulsification/solvent evaporation method. **(A)** Schematic illustration of *m*-NC structure, comprised a core-shell structure. (**B**) Cryo-TEM image of *m*-NC. (**C**) Magnetizations curve of the as-received SPIONs and a representative *m*-NCs as a function of field measured at 300 K. The saturation magnetizations of both samples were ca. 72 emu/g_{Fe}. (**D**) Transmission Mössbauer spectra of the as-received SPIONs and a representative *m*-NCs recorded at room temperature. The mean isomer shift (spectral centroid) of the SPION and *m*-NCs was 0.38 and 0.39 mm/s, respectively, is characteristic of a material comprised largely of ferric ions. "Adapted with permission from (DOI: 10.1021/acs.nanolett.6b02261). Copyright (2016) American Chemical Society."

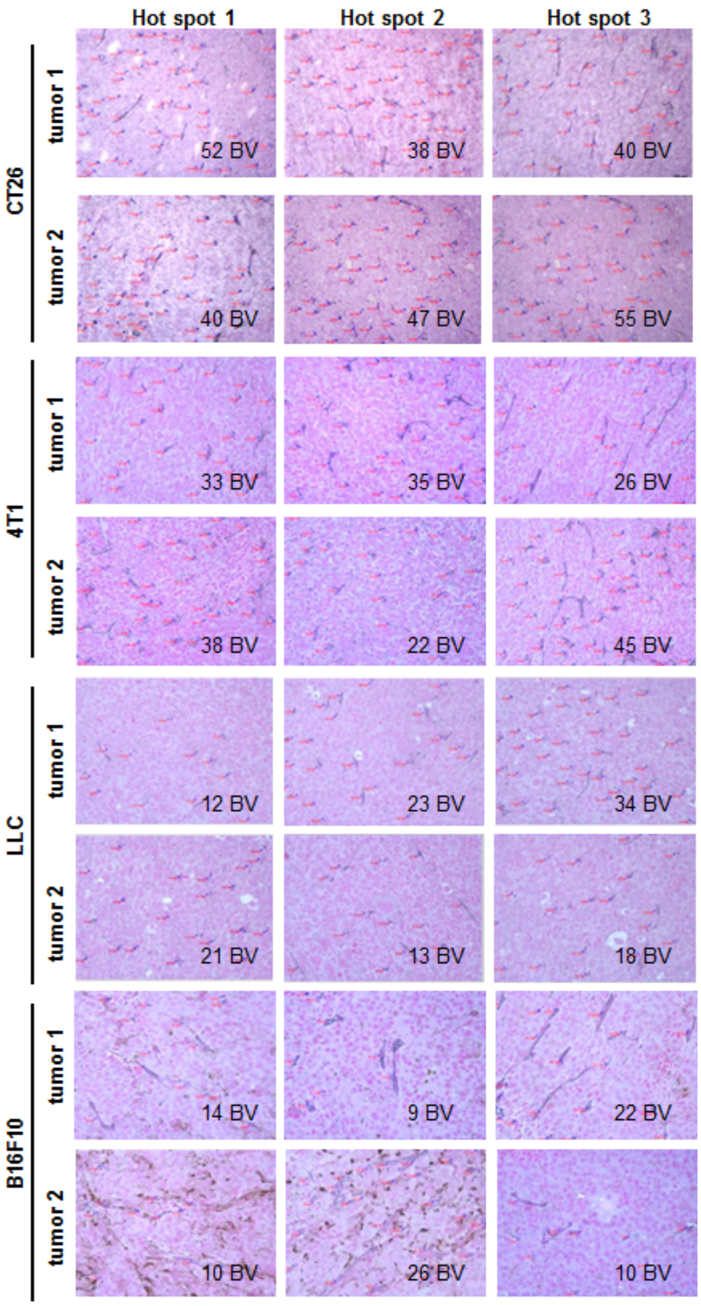

Figure S2. Microvessel density counting in four different murine tumor models. CT26 (colon), 4T1 (breast), Lewis lung carcinoma (LLC, lung) and B16F10 (melanoma) tumor tissues were excised when the volume reached 400-600 mm^3 and the tumor sections were immune-stained for CD31. Two tumor sections from two animals were included for each type of tumor. Three hot spots were selected from each section. The number of blood vessels (BV) were counted manually at 20X magnification.

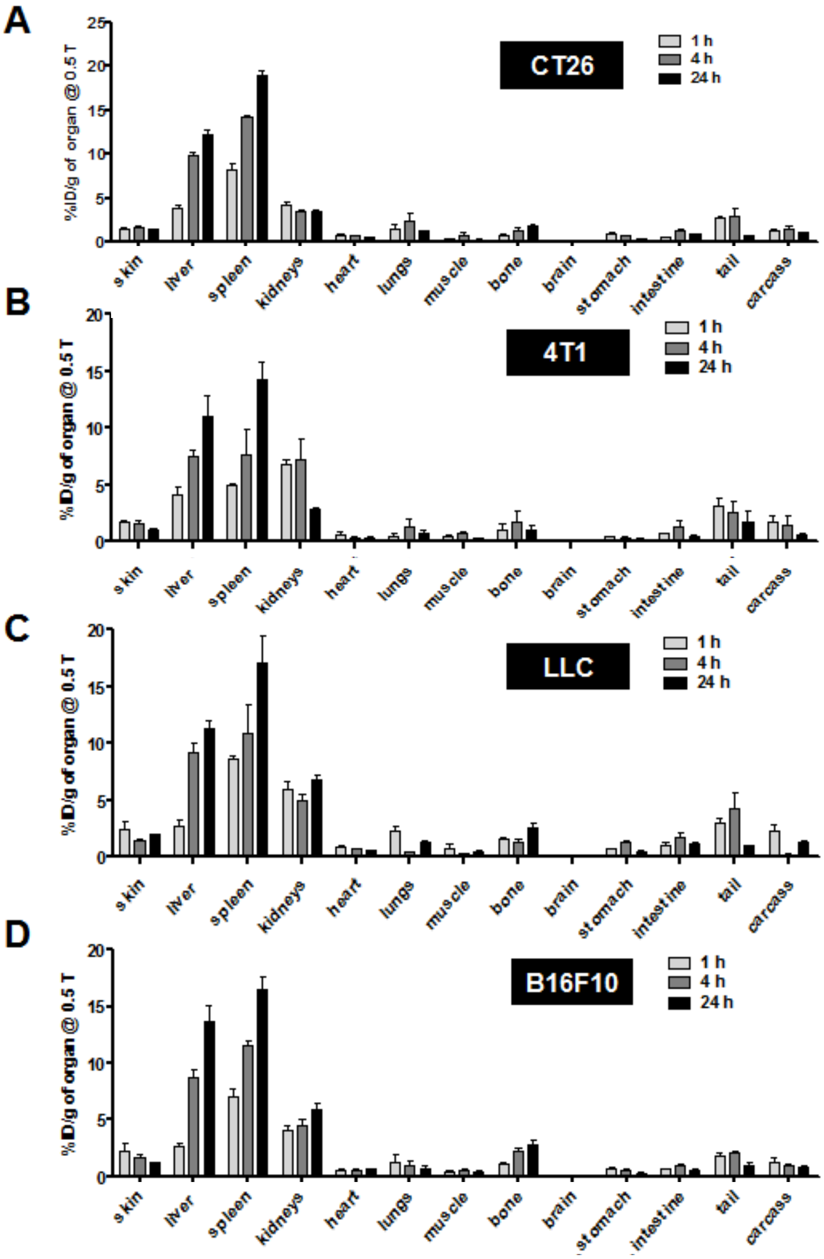

Figure S3. Organ biodistribution studies of radiolabeled magnetic nanocapsules in major organs following intravenous administration. Bifocal CT26 **(A)**, 4T1 **(B)**, LLC **(C)** and B16F10 **(D)** tumor-bearing mice were intravenously injected with *m*-NC-^{111}In at a dose of 312.5 mg polymer/kg, 125 mg SPION/kg (70 mg Fe/kg). A permanent magnet (0.43 T, 8 mm in diameter) was applied at one tumor (TU+) for 1 h. The contralateral tumor remained unexposed (TU-) and was used as a baseline control, for each of the tumor types tested. Mice were sacrificed at the specified time point, following whole body saline perfusion. Organ biodistribution profiles at 1, 4 and 24 h post-injection. Results are expressed as mean ± SEM (n = 3).

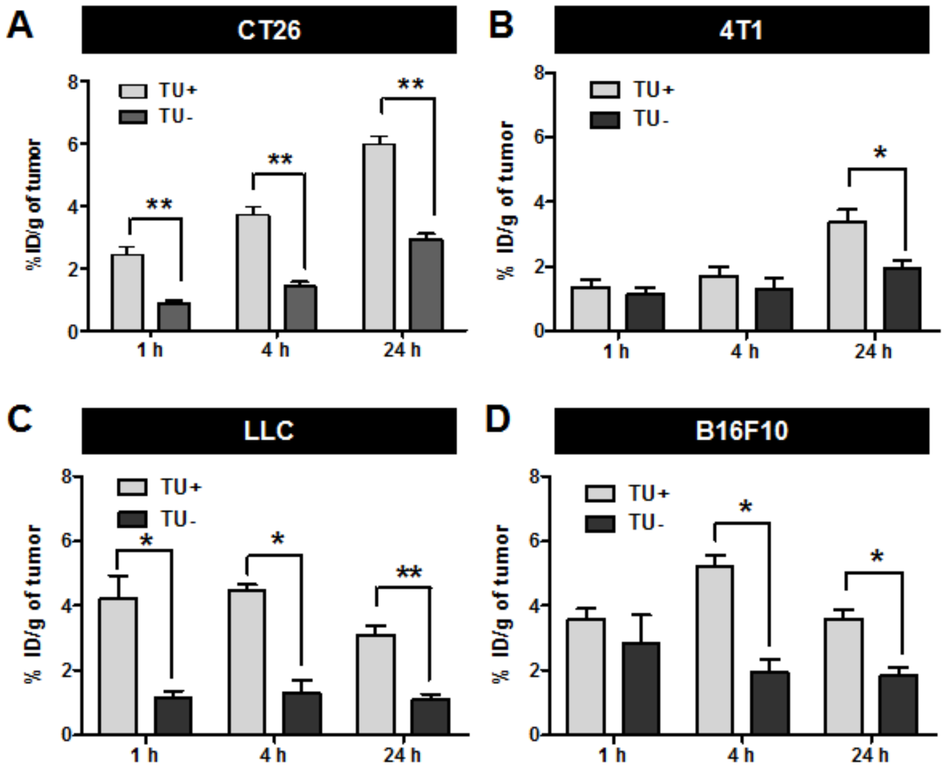

Figure S4. Passive and magnetic targeting of solid tumors *in vivo*. Bifocal CT26 **(A)**, 4T1 **(B)**, LLC **(C)** and B16F10 **(D)** tumor-bearing mice were intravenously injected with *m*-NC-^{111}In at a dose of 312.5 mg polymer/kg, 125 mg SPION/kg (70 mg Fe/kg). A permanent magnet (0.43 T, 8 mm in diameter) was applied at one tumor (TU+) for 1 h. The contralateral tumor remained unexposed (TU-) and was used as a baseline control, for each of the tumor types tested. Mice were sacrificed at the specified time point, following whole body saline perfusion. The percentage injection dose per gram tumors (%ID/g), with (TU+) or without (TU-) exposure of a magnetic field, was assessed with gamma counting, at 1, 4 and 24 h. Results are expressed as mean ± SEM (n = 3). One-way ANOVA was performed using IBM SPSS version 20 followed by Tukey's multiple comparison test (*p > 0.05) and ** p < 0.01).

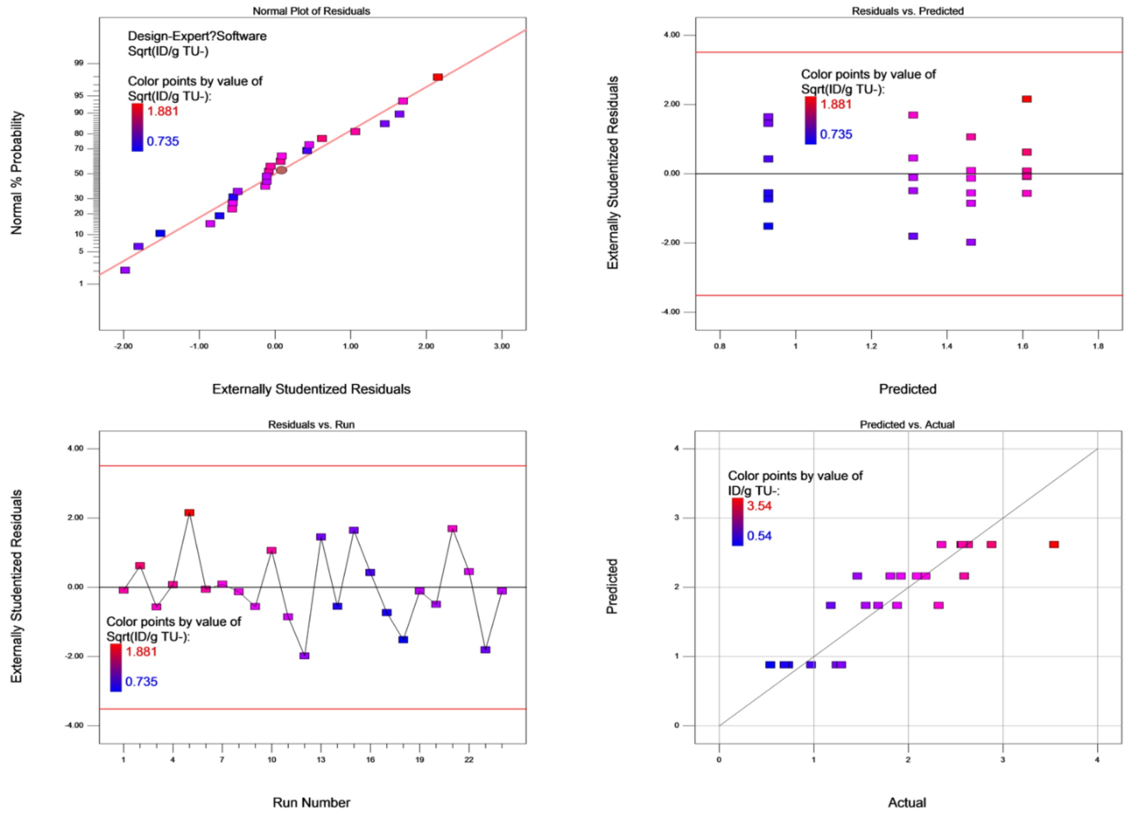

Figure S5. Normality checks for the residuals for Response 1.

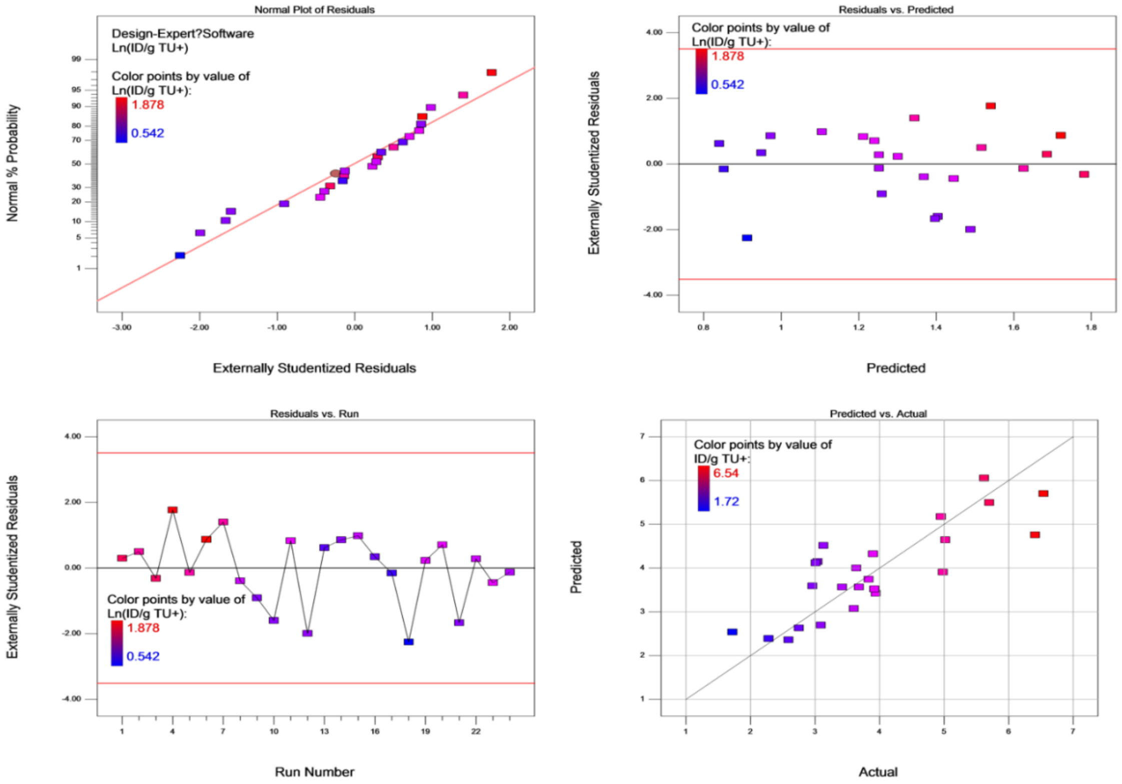

Figure S6. Normality checks for the residuals of Response 2.

Supplementary Tables

Table S1. Tumor vascularization characteristics and tumor accumulation of *m*-NCs, with and without magnetic targeting, in four murine tumor models for DoE studies

Run#	Factor A[a]	Factor B[b]	Factor C[c]	Response 1[d]	Response 2[e]
1	347	260	14.3	2.56	5.70
2	253	260	14.3	2.88	5.02
3	400	260	14.3	2.35	5.62
4	267	260	14.3	2.63	6.41
5	313	260	14.3	3.54	4.95
6	353	260	14.3	2.57	6.54
7	220	198	10.4	2.18	4.98
8	233	198	10.4	2.09	3.64
9	173	198	10.4	1.92	2.96
10	253	198	10.4	2.59	3.04
11	147	198	10.4	1.81	3.94
12	300	198	10.4	1.46	3.13
13	80	60	12.3	1.24	2.59
14	153	60	12.3	0.73	3.09
15	227	60	12.3	1.29	3.6
16	140	60	12.3	0.97	2.75
17	87	60	12.3	0.69	2.28
18	120	60	12.3	0.54	1.72
19	93	300	28.0	1.68	3.83
20	60	300	28.0	1.55	3.92
21	147	300	28.0	2.32	3.01
22	67	300	28.0	1.88	3.68
23	173	300	28.0	1.18	3.90
24	67	300	28.0	1.68	3.42

[a] MVD/microvessel density (number/mm^2)
[b] CO/blood vessel pore size cutoff (nm) as reported in the literature
[c] DM/mean blood vessel diameter (μm) as reported in the literature
[d] ID/g TU- (% injected dose/g of tumor tissue without magnetic targeting)
[e] ID/g TU+ (% injected dose/g of tumor tissue with magnetic targeting)

Table S2. Analysis of Variance Table for Response 1 (ID/g TU-)

Source	Sum of Squares	df	Mean Square	F Value	*p*-value (Prob>F)	
Model	1.55	2	0.78	37.88	<0.0001	*Significant*
Factor B (pore size)	1.55	1	1.55	75.75	<0.0001	
Factor C (diameter)	0.64	1	0.64	31.25	<0.0001	
Residual	0.43	21	0.021			
Lack of Fit	0.43	20	0.021	7.61	0.2792	*Not significant*
Pure Error	2.8E-003	1	2.8E-003			
Cor. Total	1.98	23				

Table S3. Sequential Model Sum of Squares for Response 1 (ID/g TU-)[a]

Source	Sum of Squares	df	Mean Square	F Value	*p*-value (Prob>F)	
Mean vs Total	42.350	1	42.350			
Linear vs Mean	1.580	4	0.390	18.47	<0.0001	*Suggested*
2FI[b] vs Linear	0.099	6	0.017	0.70	0.6556	
Quadratic vs 2FI	0.027	1	0.027	1.15	0.3049	*Aliased*
Residual	0.280	12	0.023			
Total	42.330	24	1.850			

[a] Highest order polynomial was selected where the model is not aliased, and the additional terms are significant.
[b] Two -factor interaction (2FI)

Table S4. Lack of Fit Tests for Response 1 (ID/g TU-)

Source	Sum of Squares	df	Mean Square	F Value	*p*-value (Prob>F)	
Linear	0.40	18	0.022	7.97	0.2727	*Suggested*
2FI	0.30	12	0.025	9.02	0.2551	
Quadratic	0.28	11	0.025	8.91	0.2553	*Aliased*
Pure Error	2.811E-003	1	2.811E-003			

Table S5. Model Summary Statistics for Response 1 (ID/g TU-)[a]

Source	Std. Dev.	R^2	Adjusted R^2	Predicted R^2	PRESS[b]	
Linear	0.15	0.7954	0.7524	0.6741	0.65	*Suggested*
2FI	0.15	0.7264	0.7264	0.2085	1.57	
Quadratic	0.15	0.7295	0.7295	0.1407	1.71	*Aliased*

[a] Model aim: to maximise the "Adjusted R^2" and the "Predicted R^2"
[b] Predicted Residual Sum of Squares (PRESS)

Table S6. Fit Summary for Response 1 (ID/g TU-)

Source	Sequential *p*-value	Lack of Fit *p*-value	Adjusted R^2	Predicted R^2	
Linear	<0.0001	0.2727	0.7524	0.6741	*Suggested*
2FI	0.6556	0.2551	0.7264	0.2085	
Quadratic	0.3049	0.2553	0.7295	0.1407	*Aliased*

Table S7. Final validation of the predicted model using R^2 press and adequate precision of response 1

Std. Dev.	Mean	C.V.%	PRESS	R^2	Adj. R^2	Pred. R^2	Adeq. Precision
0.14	1.33	10.78	0.57	0.7830	0.7623	0.7129	13.494

The "Pred R-Squared" of 0.7129 is in reasonable agreement with the "Adj R-Squared" of 0.7623; *i.e.* the difference is less than 0.2.
"Adeq Precision" measures the signal-to-noise ratio. A ratio greater than 4 is desirable. A ratio of 13.494 indicates an adequate signal. This model can be used to navigate the design space.

Table S8. Coefficient estimation of response 1

Factor	Coefficient Estimate	df	Std. Error	95% CI[a] Low	95% CI High	VIF[b]
Intercept	1.14	1	0.039	1.06	1.22	
Factor B (pore size)	0.43	1	0.050	0.33	0.54	1.67
Factor C (diameter)	-0.26	1	0.047	-0.36	-0.16	1.67

[a] Confidence Interval (CI)
[b] Variance Inflation Factors (VIF) represents the degree of the model coefficient variance that is increased due to the lack of orthogonality in the design.

Table S9. Analysis of Variance Table for Response 2 (ID/g TU+)

Source	Sum of Squares	df	Mean Square	F Value	p-value (Prob>F)	
Model	1.69	2	0.85	21.97	<0.0001	*Significant*
Factor A (density)	0.74	1	0.74	19.19	0.0003	
Factor B (pore size)	0.64	1	0.64	16.57	0.0005	
Residual	0.81	21	0.039			
Lack of Fit	0.81	20	0.040	15.01	0.2010	*Not significant*
Pure Error	2.684E-003	1	2.684E-003			
Cor. Total	2.50	23				

Table S10. Sequential Model Sum of Squares for Response 2 (ID/g TU+)[a]

Source	Sum of Squares	df	Mean Square	F Value	p-value (Prob>F)	
Mean vs Total	41.250	1	41.250			
Linear vs Mean	1.730	4	0.430	10.76	<0.0001	*Suggested*
2FI[b] vs Linear	0.350	6	0.059	1.85	0.1656	
Quadratic vs 2FI	0.013	1	0.013	0.40	0.5405	*Aliased*
Residual	0.400	12	0.033			
Total	43.750	24	1.820			

[a] Highest order polynomial was selected where the model is not aliased, and the additional terms are significant.
[b] Two-factor interaction (2FI)

Table S11. Lack of Fit Tests for Response 2 (ID/g TU+)

Source	Sum of Squares	df	Mean Square	F Value	p-value (Prob>F)	
Linear	0.76	18	0.042	15.79	0.1958	*Suggested*
2FI	0.41	12	0.034	12.74	0.2159	
Quadratic	0.39	11	0.036	13.45	0.2099	*Aliased*
Pure Error	2.684E-003	1	2.664E-003			

Table S12. Model Summary Statistics for Response 2 (ID/g TU+)[a]

Source	Std. Dev.	R^2	Adjusted R^2	Predicted R^2	PRESS[b]	
Linear	0.20	0.6937	0.6292	0.5382	1.18	*Suggested*
2FI	0.18	0.8348	0.7077	0.4177	1.46	
Quadratic	0.18	0.8401	0.6935	0.3422	1.64	*Aliased*

[a] Model aim: to maximize the "Adjusted R^2" and the "Predicted R^2"
[b] Predicted Residual Sum of Squares (PRESS)

Table S13. Fit Summary for Response 2 (ID/g TU+)

Source	Sequential *p*-value	Lack of Fit *p*-value	Adjusted R^2	Predicted R^2	
Linear	<0.0001	0.1958	0.6292	0.5282	*Suggested*
2FI	0.1786	0.2159	0.7077	0.4177	
Quadratic	0.4615	0.2099	0.6935	0.3422	*Aliased*

Table S14. Final validation of the predicted model using R^2 press and adequate precision of response 2

Std. Dev.	Mean	C.V.%	PRESS	R^2	Adj. R^2	Pred. R^2	Adeq. Precision
0.20	1.31	14.97	1	0.6766	0.6458	0.5951	13.631

The "Pred R-Squared" of 0.5951 is in reasonable agreement with the "Adj R-Squared" of 0.6458; *i.e.* the difference is less than 0.2.

"Adeq Precision" measures the signal-to-noise ratio. A ratio greater than 4 is desirable. A ratio of 13.631 indicates an adequate signal. This model can be used to navigate the design space.

Table S15. Coefficient estimation of response 2

Factor	Coefficient Estimate	df	Std. Error	95% CI[a] Low	95% CI High	VIF[b]
Intercept	1.33	1	0.045	1.24	1.42	
Factor B (pore size)	0.31	1	0.070	0.16	0.46	1.04
Factor C (diameter)	0.22	1	0.054	0.11	0.33	1.04

[a] Confidence Interval (CI)

[b] Variance Inflation Factors (VIF) represents the degree of the model coefficient variance that is increased due to the lack of orthogonality in the design.
